# Diagnostic Challenges in Chondroblastic Maxillary Osteosarcoma: A Case Report

**DOI:** 10.7759/cureus.8418

**Published:** 2020-06-02

**Authors:** Mohannad K Rajab, Baraa I Awad, Hadi Afandi Al-Hakami, Haneen Al-Maghrabi

**Affiliations:** 1 Otolaryngology-Head and Neck Surgery, Ministry of Health, Makkah, SAU; 2 Otolaryngology-Head and Neck Surgery, King Saud bin Abdulaziz University for Health Sciences, King Abdullah International Medical Research Center, Jeddah, SAU; 3 Otolaryngology, King Saud bin Abdulaziz University for Health Sciences, King Abdullah International Medical Research Center, Ministry of National Guard Health Affairs, Jeddah, SAU; 4 Pathology, King Faisal Specialist Hospital and Research Center, Jeddah, SAU

**Keywords:** osteosarcoma, craniofacial, chondroblastic, maxillary

## Abstract

The diagnosis of craniofacial osteosarcoma can be quite challenging, and the condition often goes unrecognized for a considerable period of time. In this report, we discuss the case of a 21-year old woman who presented with a one-year history of a small swelling over the left maxillary alveolar ridge. Upon further investigation, the histopathological examination showed high-grade chondroblastic osteosarcoma. The option of four cycles of neoadjuvant chemotherapy regimen preoperatively was chosen, and left inferior maxillectomy was performed along with reconstruction with obturator prosthesis. This case highlights the difficulties encountered in such rare cases of craniofacial osteosarcomas both in terms of the delay in the establishment of the diagnosis as well as management protocol. A high index of suspicion is required in cases of craniofacial osteosarcoma and early surgical resection with adequate safety margins is warranted.

## Introduction

Osteosarcoma, also called osteogenic sarcoma, is a rare, aggressive, and malignant form of tumor, and it is considered to be the most common primary malignant bone tumor [1]. Osteosarcoma accounts for around 20% of all sarcomas, and the majority of osteosarcoma cases are found to be primary and affect adolescents or young adults [2,3]. Secondary osteosarcoma tends to affect older groups, usually during their fifth or sixth decade of life, and it appears either as a transformation of an existing bone condition like Paget’s disease or is caused due to irradiation [3,4]. It has a predilection for long bones and typically arises in metaphyses of long bones, with the most common sites being the distal femur, proximal humerus parts, and proximal tibia [3,5].

A tumor is defined as craniofacial if it occurs in the jaw, maxilla, or other unspecified regions [6]. Craniofacial osteosarcoma accounts for only 1% of all head and neck malignancies with a very rare incidence rate between 2-13% of all osteosarcomas, while osteosarcoma of the jaw represents around 6-9% of all osteosarcoma cases [7-10]. It tends to present later in life compared to the osteosarcoma of long bones, typically in the third or fourth decade [3]. Meanwhile, maxillary tumors are often seen in the inferior regions, such as the alveolar ridge, sinus floor, and palate, rather than the superior parts such as zygoma and orbit [3,10]. Moreover, presenting signs and symptoms can include regional swelling, pain, paresthesia, changes in teeth positions, or even loss of teeth [3,7]. Regarding radiological features, it includes the incidental discovery of an opacity, radiolucency, bone irregularity, or a widened periodontal ligament (PDL) space [10]. Due to the non-specificity of these symptoms and their overlap with multiple etiologies such as infectious and developmental conditions, the diagnosis is usually considerably delayed [3,11]. Furthermore, because of the rarity of such tumors, it is often difficult to assess treatment protocols and prognosis. Thus, osteosarcoma of the jaw often presents a challenging situation for clinicians.

The modern treatment protocol for osteosarcoma is multimodal, consisting of preoperative chemotherapy followed by extensive surgery and postoperative chemotherapy [12]. With regard to medication used for chemotherapy, they are high-dose combinations of methotrexate, cisplatin, doxorubicin, and/or ifosfamide. Therefore, early diagnosis and adequate surgical resection are the keys to high survival rates.

Our objective in preparing this case report was to highlight the diagnostic difficulties encountered in a patient with osteosarcoma that presented as small mass over the left maxillary alveolar ridge.

## Case presentation

A 21-year-old female, a Saudi student, presented to the Otolaryngology Department as an outpatient complaining of a left upper maxillary alveolar progressive recurrent mass of six months. The mass had started as a small swelling over the left maxillary alveolar ridge where excision had been done accordingly in the dental office. One month later, the mass had reappeared with rapid growth and had never regressed until a facial bulge was visible. On further history, the patient did not mention any problems with swallowing or respiration, and her past medical history was insignificant with no chronic illness and no prior history of exposure to radiation or chemotherapy and no history of benign bone conditions. Regarding her family history, there was no genetic predisposition or other malignancies. Furthermore, surgical and dental history was insignificant with no pharmacological medications and no history of allergies or smoking.

**Figure 1:**
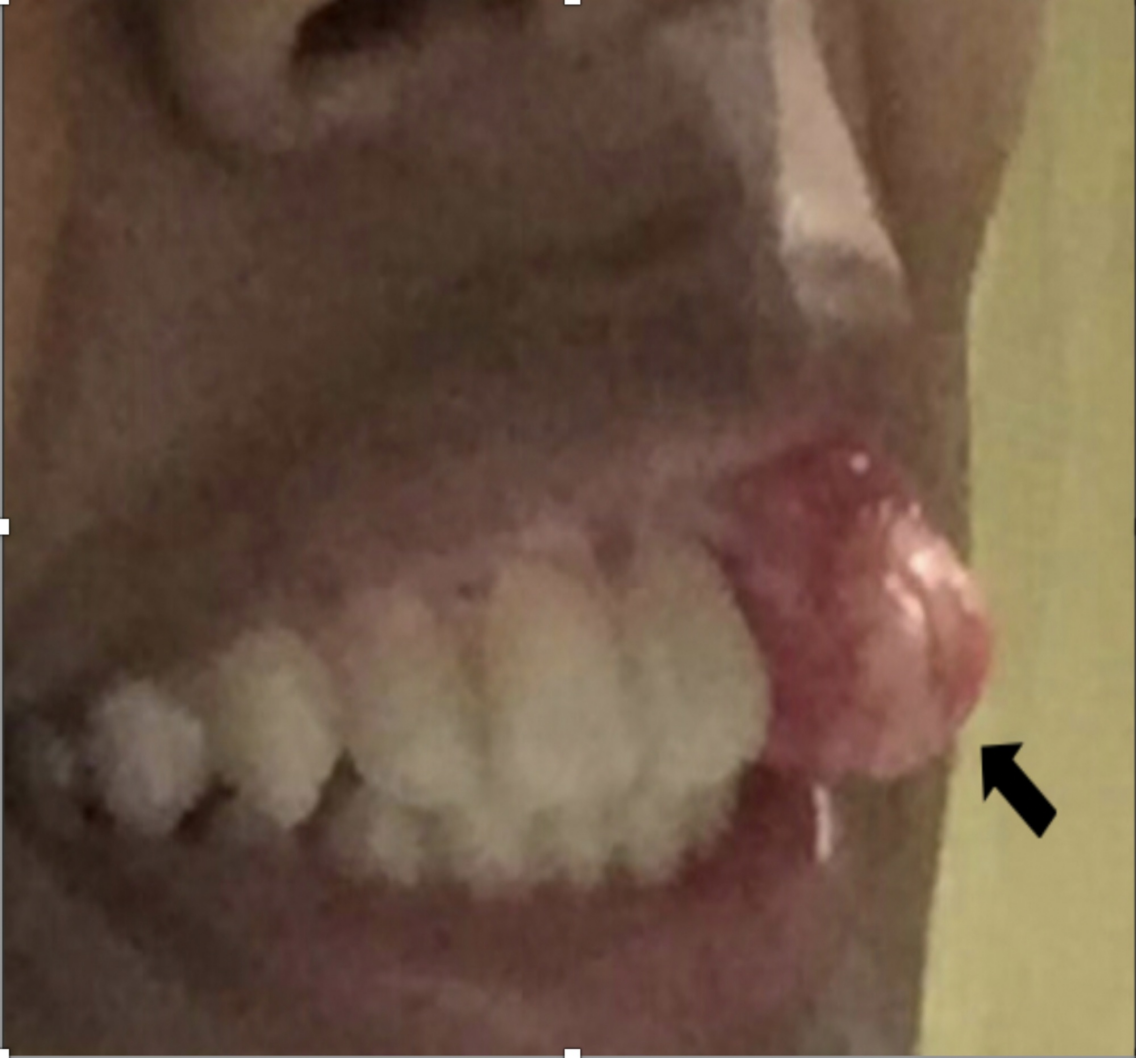
Picture of the lesion at the time of the presentation Irregular mass over the left anterior maxillary alveolar ridge with missed upper lateral incisor was noted (black arrow)

On physical examination, an irregular mass on the left anterior maxillary alveolar ridge measuring around 2 x 2 cm (Figure 1) with a missed upper lateral incisor was noted. The overlying mucosa was intact and there was no ulceration. There was no pus coming out of the mass and examination of the cervical lymph nodes revealed no abnormalities. Upon radiological workup, there was no evidence of distant metastasis or any signs of other abnormality.

**Figure 2:**
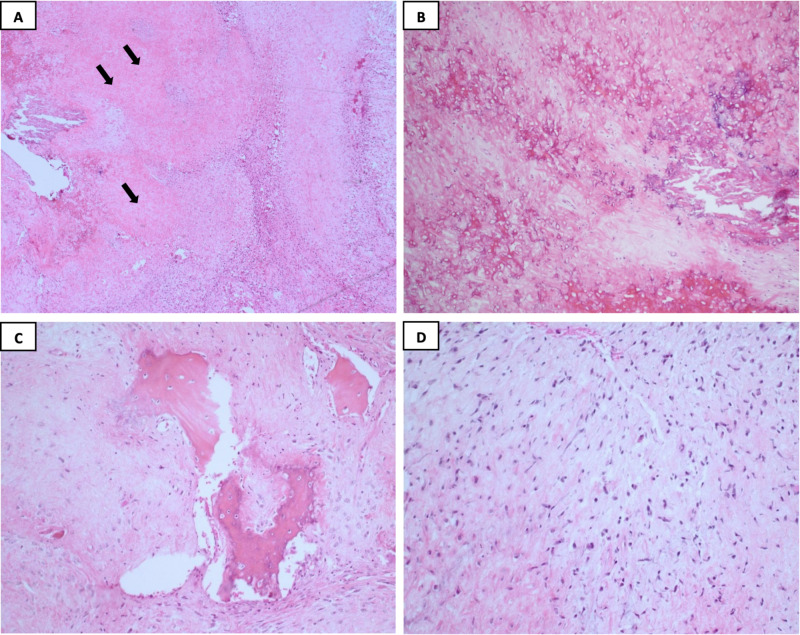
Histopathological examination of the biopsy The histopathological report of the biopsy revealed fragments of large infiltrative lobules destroying the bone composed of the central hypocellular area (black arrows; 2A and 2C) containing chondroid and osteoid tissue with focal necrosis surrounded by myxoid background (2B). Moreover, the neoplastic cell characteristics are ovoid to spindle with moderately irregular nuclear contours, and contain medium size nucleoli, arranged haphazardly in a myxoid background (2D). Meanwhile, numerous mitotic figures were also seen

The recurrence of the mass warranted further investigation as an initial differential of osteomyelitis or other mixed lesions. After obtaining the patient’s written consent, an incisional biopsy was performed under local anaesthesia where the histopathology showed high-grade (G3), chondroblastic osteosarcoma (Figure 2).

When we discussed the surgical options and treatment with the patient, she was reluctant to follow the treatment protocol and chose to seek a second opinion. After a few months, she presented with a slight increase in mass size (3 x 2 cm).

**Figure 3:**
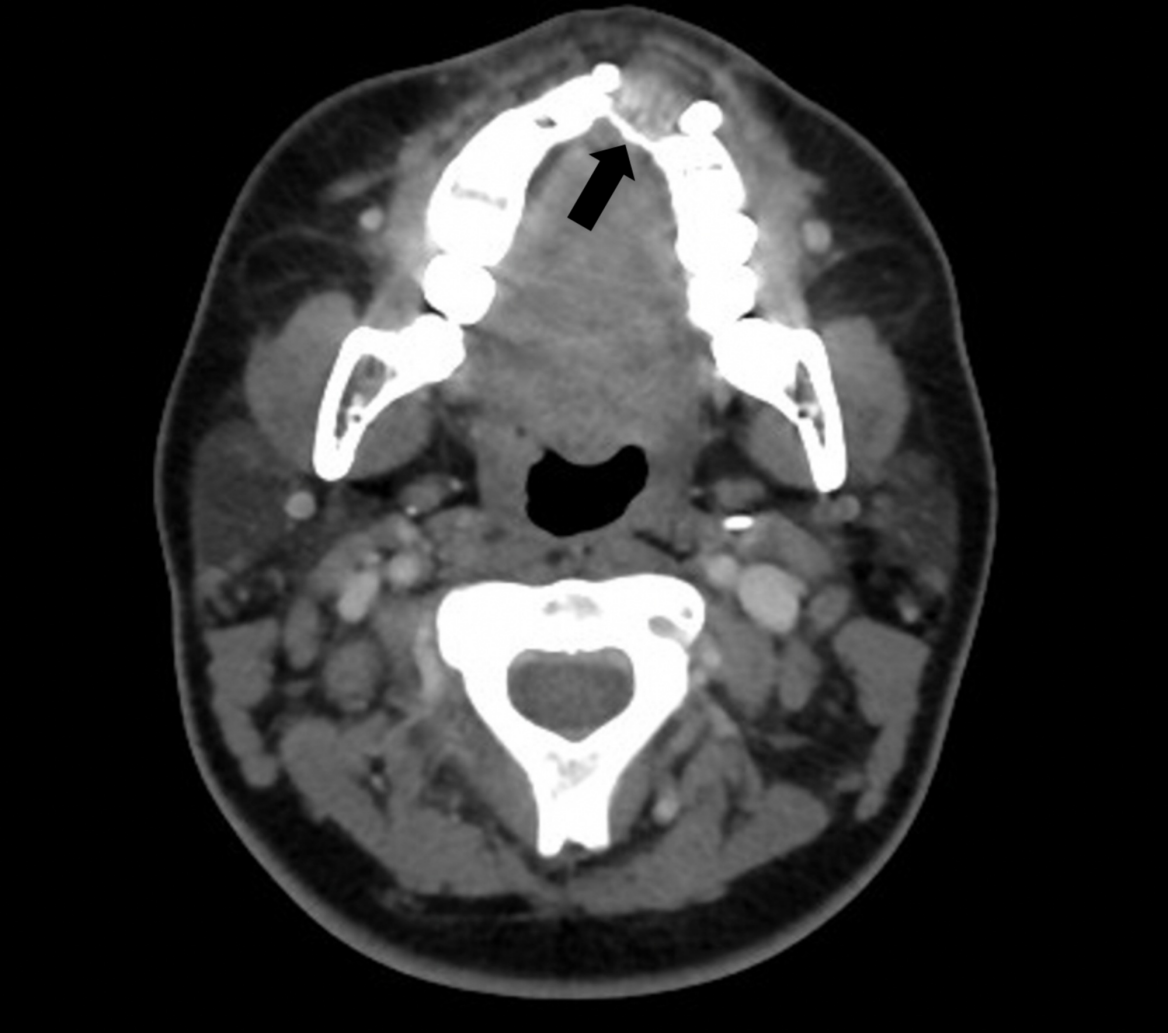
CT of the neck The lesion is seen in the alveolar process of the left maxilla and appears mostly lytic with no material nor sunburst changes while the lesion itself was seen between the left incisor and the left canine (black arrow); meanwhile, the left lateral incisor was not seen CT: computed tomography

**Figure 4:**
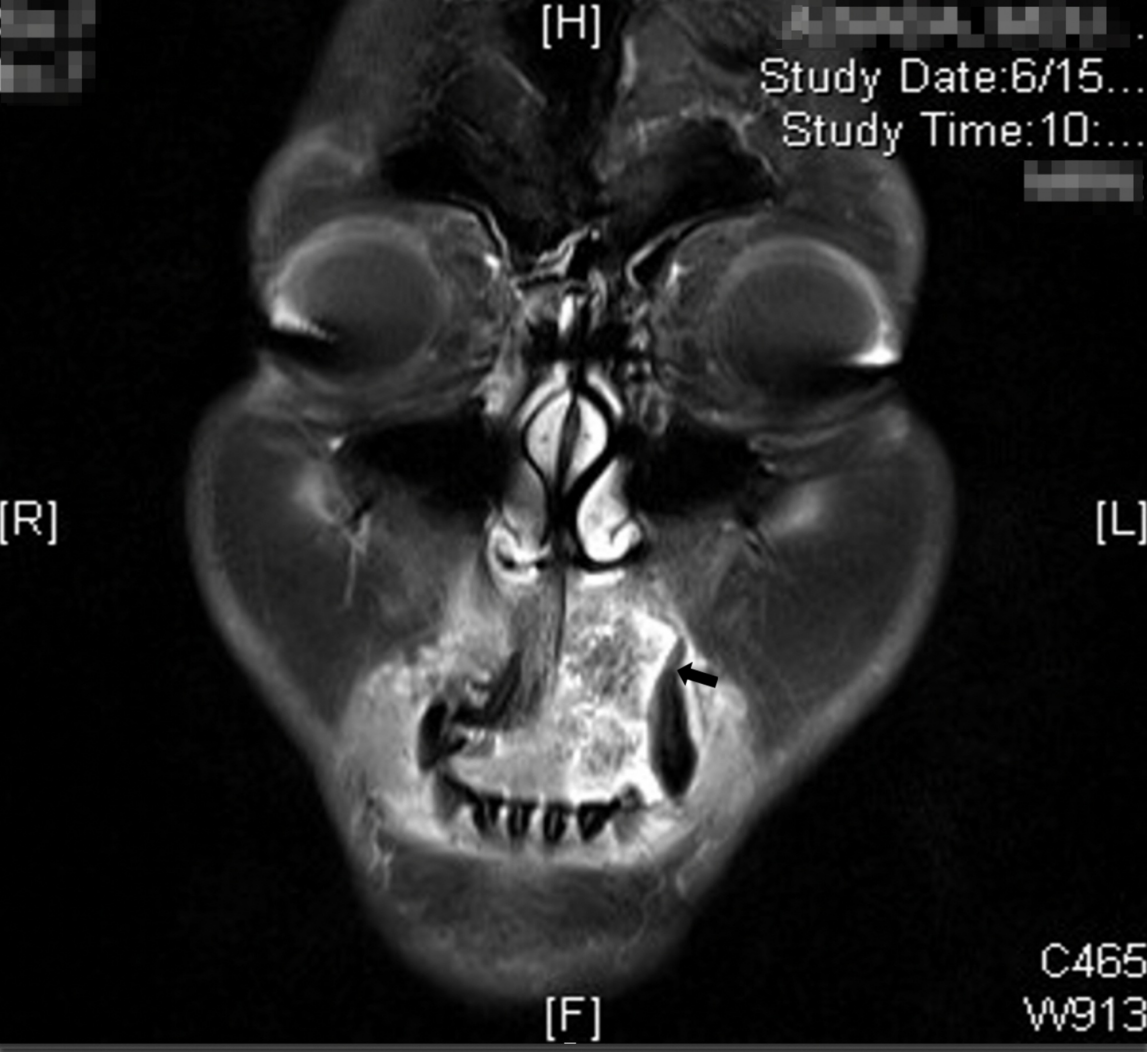
MRI of the neck Left maxillary alveolar ridge osteolytic soft tissue mass of around 3.3 x 1.8 cm extending superiorly to the inferior nasal apparatus (black arrow) MRI: magnetic resonance imaging

A CT scan of the neck done on the same day showed a maxillary lesion with small growth (3 mm). The lesion was seen in the alveolar process of the left maxilla and appeared to be mostly lytic with no material or sunburst changes, while the lesion itself was seen between the left incisor and the left canine; meanwhile, the left lateral incisor was not seen (Figure 3). Also, there was no extension into the anterior nasal septum or incisive canal, no superior extension to the submucosa of the left nasal cavity, and no invasion into the palate. The lesion was separate from the orbicularis aureus muscle with no infiltration of the skin and subcutaneous tissue. In addition, a diffuse bilateral mildly enlarged lymph node was noted at level 2A with a few subcentimetric occipital lymph nodes. The adenoid was enlarged, causing mild narrowing of the airway with no obstruction whatsoever. Further investigation was requested, and an MRI of the neck showed left maxillary alveolar ridge osteolytic soft tissue mass of around 3.3 x 1.8 cm extending superiorly to the inferior nasal apparatus (Figure 4). Meanwhile, CT scans of chest, abdomen, and pelvis were ordered to exclude any other malignancies, and they showed no evidence of metastasis or lesions.

**Figure 5:**
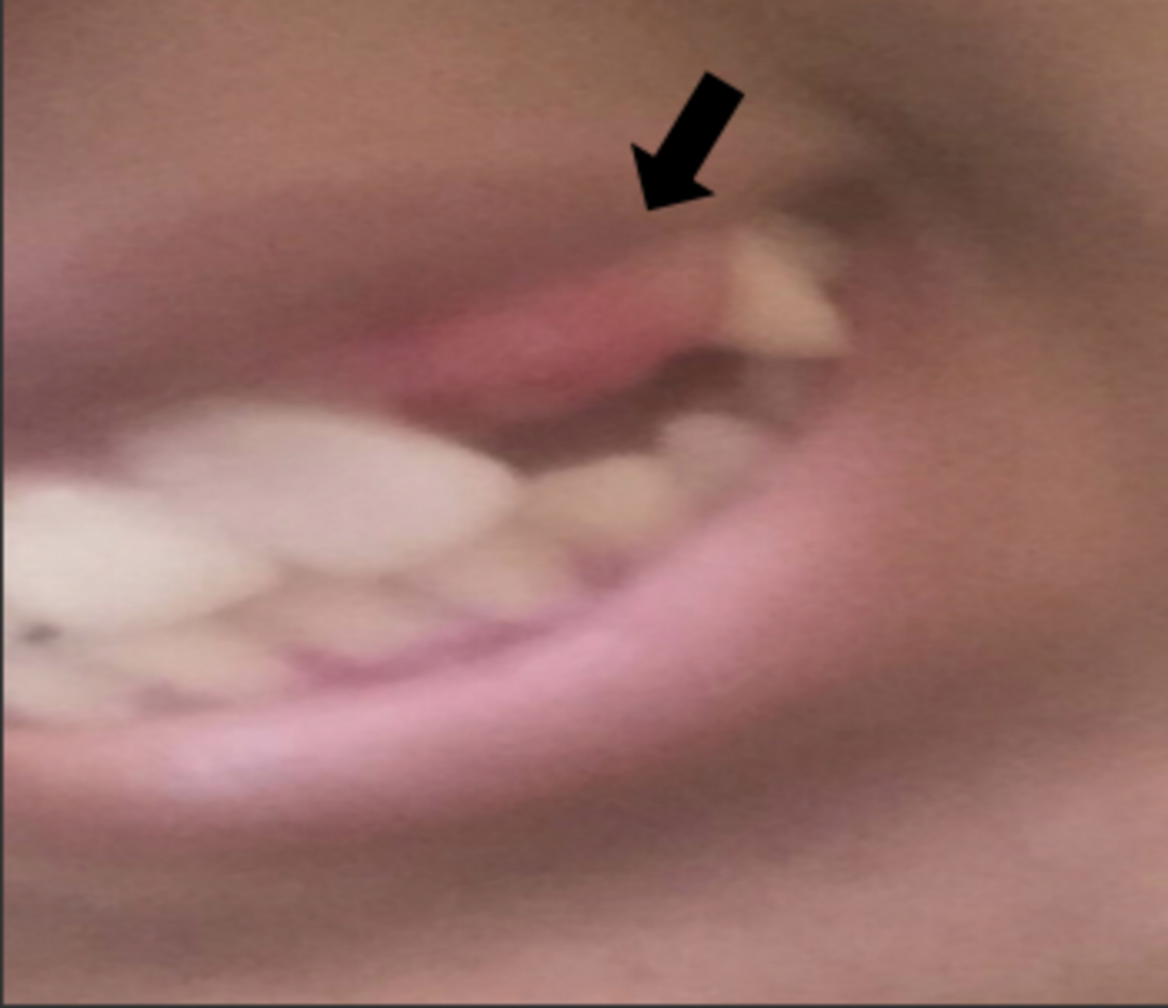
Picture of the lesion after neoadjuvant chemotherapy The response to the chemotherapy was very good and the tumor size shrank substantially (black arrow)

Regarding the treatment regimen, the case was discussed in the tumor board and four cycles of neoadjuvant chemotherapy were given, which consisted of doxorubicin and cisplatin. The response to the chemotherapy was very good and the tumor size shrank substantially (Figure 5). The chemotherapy was followed by left inferior maxillectomy with a 1.5-cm safety margin with tracheostomy and a left-sided supra-omohyoid neck dissection. Intraoperatively, the frozen section was taken post excision, which showed negative safety margins. With regard to reconstruction, it was carried by an obturator prosthesis, which was planned due to the unavailability of the plastic surgeon. Postoperatively, the patient was admitted to the ICU for a few days for close observation of respiration and, one week later, she was discharged home in stable condition and was advised to follow up with the Oncology Centre for adjuvant chemotherapy.

**Figure 6:**
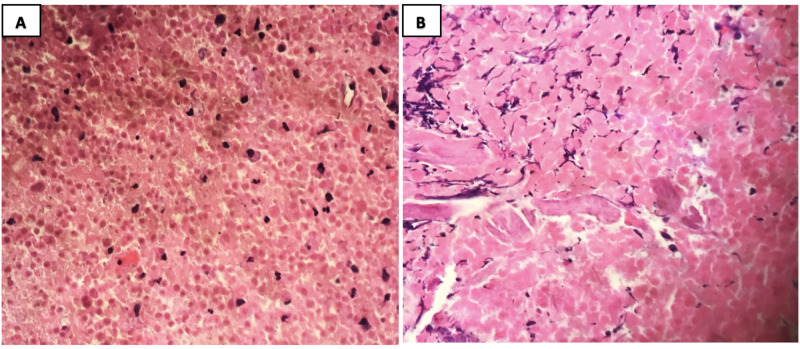
Histopathology examination of tumor section post-therapy using hematoxylin and eosin (H&E) stain A: high-power examination shows complete tumor necrosis; residual inflammatory cells are seen in the background (H&E; 40x). B: remnants of acellular osteoid, tumor necrosis, and fibrosis (H&E; 40x)

The final histopathology showed a tumor that measured 3.5 x 2.5 x 2.0 cm with extensive therapy-related effect (>99% necrosis) (Figure 6) and negative margins. There was no lymphovascular invasion and all the resected lymph nodes were negative for malignancy. The final pathological staging post-surgery was T0N0M0. The patient received two further cycles of doxorubicin and cisplatin postoperatively, and she is still following up with the Oncology Center with no signs or symptoms of recurrence for three years.

## Discussion

Osteosarcomas are a heterogeneous group of malignancies characterized by the production of osteoid or neoplastic ossification [13]. Osteosarcoma of the jaw is among the most uncommon neoplasms, and it represents around 4% of all osteosarcoma cases [14]. It tends to occur in the third or fourth decade of life while the typical osteosarcoma of long bones usually occurs in the first or second decade [15]. The rarity of these tumors and the non-specificity of the symptoms usually lead to a considerable delay in the diagnosis [3]. In our case, the presenting complaint and some demographic characteristics such as age and sex of the patient were in accordance with some studies in the literature [6,11,13]. However, diagnosis and management were challenging in different aspects. Firstly, despite the typical presentation of swelling, the diagnosis had been delayed. This is quite frequent with osteosarcoma due to the rarity of these tumors. Nevertheless, a high index of suspicion is required to make the correct diagnosis in such cases. Moreover, this case did not have any history of predisposing factors for osteosarcoma; nor was there any family history of malignancies or other inherited conditions that could be associated with osteosarcoma.

Regarding surgical resection, due to the complex anatomy of the maxillofacial region, getting negative margins in surgical resection as well as reconstruction is quite challenging [16]. Moreover, a tumor in the maxillofacial region is daunting to the patient, and it is often associated with a heavy psychological impact and fear from facial disfigurement. This can often lead to patient neglect as many of them tend to take the wait-and-see approach [17]. The use of chemotherapy before the surgery can shrink and delineate the tumor, which can contribute to facilitating the surgical resection and improving the quality and adequacy of the surgical margin without compromising the functional and the aesthetic aspect as much as possible [18]. In addition, it allows for predicting the prognosis by evaluating the response to the preoperative chemotherapy histologically [18]. In our case, there was an extensive therapy-related effect with >99% necrosis and negative margins, which indicated a good prognosis.

There are various histopathological classifications, and the subtypes are classified according to the predominant matrix produced by the tumor: osteoblastic (predominantly bone matrix), chondroblastic (predominantly cartilaginous matrix), or minimal matrix in fibroblastic [19]. Special attention must be paid in order to differentiate between two variants that can simultaneously occur in osteosarcoma of the jaw: chondroblastic osteosarcoma and chondrosarcoma [2]. According to George et al., the chondroblastic variant exhibits the worst prognosis due to the difficulty of differentiating it from chondrosarcoma [2]. A biomarker has been suggested to help differentiate between them using immunochemistry and western blot technique with galectin-1 being reported to be abundant in chondroblastic osteosarcomas but not in chondrosarcoma [20]. Although this technique was not used in our case, it is recommended to be applied in the future along with other advanced techniques in cases where there are challenges in differentiating between cartilage-producing tumors.

## Conclusions

The diagnosis of craniofacial osteosarcoma is quite challenging and needs a high index of suspicion. The mainstay of treatment in osteosarcoma cases is radical surgical resection with obtaining negative margins alongside chemotherapy, which increases early survival rates considerably, with the multimodal therapy being the most effective in increasing long-term survival rates.
